# Supplementary material for: Are carbon emissions trading and green financial instruments synergistic? -Comprehensive quantitative research based on content analysis
Source: PLoS One. 2024 Mar 7;19(3):e0298601. doi: 10.1371/journal.pone.0298601 (PMC10919601; doi:10.1371/journal.pone.0298601)
Supplement: S1 Data — (ZIP) [file pone.0298601.s001.zip › Data/地级市Z_F工作报告.zip!/╣≤╓▌/░▓╦│╩╨/└┤╫╘╣1⁄2╓┌║┼í╛┬φ┐╦╩2╛▌═°í┐_2008.docx]

各位代表:

现在，我代表市人民政府，向大会作工作报告，请予审议，并请各位政协委员和其他列席人员提出意见。

2007年政府工作回顾

2007年，市人民政府在市委的领导下，在市人大和市政协的监督、支持下，按照科学发展观和构建社会主义和谐社会的要求，紧紧围绕市第二次党代会提出的奋斗目标，坚定不移地深入实施农业稳市、旅游兴市、环境立市、工业强市战略，全面实施人才战略，优先发展科教事业，一心一意谋发展，聚精会神搞建设，与时俱进、开拓进取、团结拼搏，创造性地开展工作，经济发展呈现出速度比较快、效益比较好、结构进一步优化的特点，社会事业全面进步，人民生活得到改善，各项工作展示出又好又快发展的良好态势。

一、经济增长速度不断加快，综合经济实力明显提高

全市生产总值142.03亿元，同比增长15.1%，高于全国3.7个百分点、全省1.4个百分点，创撤地设市以来最好水平；财政总收入24.01亿元，同比增长30.6%，其中地方财政收入15.05 亿元，同比增长35.3%；全社会固定资产投资完成80.21亿元，同比增长18.3%；年末金融机构存款余额168.85亿元、贷款余额150.77亿元，分别比年初增长8.1%、14.7%；社会消费品零售总额39.52亿元，同比增长19.9%。三次产业结构调整为19.07：40.73：40.20。

二、坚持又好又快发展，经济社会发展的协调性得到增强

以路网为重点的基础设施建设进一步加强。结合编制城市发展战略规划，全面启动了城市总体规划、土地利用总体规划和城市路网规划修编工作。开工建设了一批城市道路，新增城市道路面积37.03万平方米。军民路、迎宾路、民主路、金钟广场、南华菜场完成建设改造，黄果树大街、中华东路、贯城河综合治理二期、东二环路、西航路延伸段、机场路、虹山环湖路等工程建设进展顺利，市污水处理厂、南马广场、贵州移动客户服务第二中心等项目开工建设。镇胜高速公路部分开通，黄望高速公路前期工作和黄织铁路建设进展顺利。全市1126公里通村公路和261公里通乡油路建设全面推进。各县区相继完成了一批城镇基础设施建设项目。全市实现了村村通电和村村通移动电话。

农业农村工作取得新成绩。认真贯彻落实中央、省委和市委1号文件精神，全面落实各项支农惠农政策，共发放各种支农惠农补贴资金17287.91万元。粮食产量达到78.99万吨，创历史新高。“一乡一特、一村一品”规模不断扩大，形成了一批特色明显、类型多样的专业乡镇和专业村。建设生态畜牧业强市步伐加快，畜牧业产值占农业总产值比重达46.2%。大力推进农业产业化经营，龙头企业辐射带动作用明显增强，实现了村村有协会，农民进入市场的组织化程度进一步提高。实施农村饮水安全工程和“三小工程”，完成2.66万亩烟水配套工程，解决了10.21万人的饮水安全，新增有效灌溉面积5.94万亩。扎实推进社会主义新农村建设，各级新农村建设规划全面完成，省、市、县三级新农村建设示范村工作有序推进。新阶段扶贫开发取得新成效，全市净减少低收入贫困人口12759人。编制完成了大中型水库移民后期扶持规划，发放大中型水库后期移民扶持资金2400万元，移民的生产生活条件逐步改善。

工业经济发展加快。编制完成了安顺航空城、黎阳高新技术产业园区、西秀工业园区、普定循环经济工业基地规划，一批重点项目陆续进入基地和工业园区。航空城建设顺利启动，云马飞机零部件转包等项目落户航空城。能源、装备制造、化工、药品、食品、旅游商品等优势产业进一步壮大。积极引导支持军工企业发展民品生产，军地军民结合进一步加强。全市规模以上工业实现增加值38.06亿元，同比增长16.0%。纳入地方统计的规模以上工业实现增加值24.72亿元，同比增长16.0%，实现利润总额5.43亿元，同比增长136.1%；实现税金总额5.95亿元，同比增长47.3%。

旅游业发展质量提高。我市被评为中国优秀旅游城市，黄果树、龙宫成功申报为全国首批“5A”级旅游景区，平坝县被评为中国屯堡文化之乡和中国屯堡文化研究基地。新建了一批星级酒店、商业购物中心。清镇高速公路沿线建筑景观整治取得阶段性成效。成功举办了黄果树瀑布节、油菜花旅游节、屯堡文化旅游节、旅游商品展销暨“两赛一会”、紫云格凸全国首届攀岩挑战赛等旅游节庆活动，成功承办了国务院西开办和国家旅游局主办的“大力发展旅游产业、深入推进西部大开发工作座谈会”，进一步拓宽了旅游市场，提升了安顺的知名度和美誉度。全年共接待游客960万人次，同比增长29.9%，旅游总收入54.00亿元，同比增长45.6%。在旅游业的带动下，第三产业快速发展，实现增加值57.10亿元，同比增长17.3%。

生态和环境优势进一步显现。牢固树立保住青山绿水也是政绩的发展理念，扎实推进以退耕还林、天然林保护等为重点的生态建设，共完成营造林14.43万亩，森林覆盖率达36%，城市绿化率达30%。全市所有县区均被列为全国石漠化综合治理试点县。建成沼气池20462口。完成水土流失综合治理面积58.48平方公里。万元生产总值能耗比上年降低4.62%，二氧化硫排放减少4.03%。市区和各县城区环境空气质量达到国家二级标准，城市空气质量优良率保持100%，集中式饮用水源全面达标。全市人均预期寿命达到71岁，高于全省平均水平2.38岁。

各项社会事业全面发展。“两基”成果进一步巩固。全市小学适龄儿童入学率和初中阶段毛入学率分别达98.97%和98.20%，青壮年非文盲率提高到98.67%，西秀区通过省政府“两基”评估复查验收，农村薄弱学校改造工程基本完成。高中阶段教育、高等教育、职业教育、特殊教育和民族教育等各级各类教育协调发展。安顺市民族中学被评为省级三类示范性高中。制定并实施了安顺市科技发展规划，科技进步对经济增长的促进作用得到加强。认真开展专利执法和知识产权保护，完成了农特产品地理标志普查工作。文物和非物质文化遗产保护得到加强，西秀区宁谷牂牁郡遗址被确认为贵州省第一个中央集权地方政府，填补了贵州地域和历史的空白，使安顺在贵州历史上的地位和作用得到进一步的肯定。新建成农村广播电视“村村通”工程261座，完成了卫星广播电视地面接收设施转星工作。医疗卫生服务能力得到提高，城市社区和县乡村三级卫生服务体系进一步完善，重大传染病、地方病防治工作力度加大。人口和计划生育工作新机制建设取得成效，人口出生率、人口自然增长率分别为13.01‰和6.37‰，同比下降0.19和0.73个千分点。全民健身活动蓬勃开展，我市选手在全国第八届少数民族运动会上取得“三金一银”的好成绩。全面落实党的民族宗教政策，民族团结，宗教稳定。国防建设与经济建设协调发展，国防动员工作有新提高。人事人才工作进一步加强。妇女儿童、老龄和残疾人事业健康发展。档案、史志、人防、外事侨务、对台等工作取得新成绩。

三、解决事关民生问题的力度进一步加大，人民群众得到更多实惠

城镇就业再就业、就业培训、劳务输出等目标任务超额完成，对“零就业家庭”等就业困难群众给予了重点帮扶，全年新增就业再就业15027人，城镇登记失业率控制在4.36%。基本养老、基本医疗、失业、工伤和生育等社会保险覆盖面进一步扩大，保障标准进一步提高。启动了农民工医疗保险工作。建立农村最低生活保障制度，共将13.5万农村贫困人口纳入低保范围，发放低保资金2428.18万元，开创了从制度上抑制贫困的新格局。提高了城市低保标准，共发放低保金4105.86万元、物价补贴537.7万元。调增了企业退休人员基本养老金。新型农村合作医疗实现全覆盖，参合率达85.27%。全面落实农村义务教育“两免一补”政策，共落实资金7428万元，惠及学生82.44万人次。城乡居民收入水平和生活质量得到提高，城镇居民人均可支配收入突破万元，达到11109元，实际增长22.6%；农民人均纯收入2379元，比上年增加近400元，实际增长11.9%。认真落实“米袋子”、“菜篮子”行政首长负责制，市场供应充足，物价稳定，产品质量和食品安全专项整治工作取得阶段性成效。启动学有所教、劳有所得、病有所医、老有所养、住有所居行动计划。制定了《安顺市2007—2011年廉租住房保障实施意见》，完成了第二期廉租房建设任务并租赁给符合条件的城市低收入住房困难家庭，向城市低收入住房困难户发放了房屋租赁补贴和实行租金核减。东关经济适用住房建设取得新进展。土地征用、房屋拆迁等带来的涉及人民群众切身利益的突出问题正在逐步得到解决。“十件实事”总体完成。

四、改革开放深入推进，发展活力进一步增强

完成了安顺试验区申报国家综合配套改革试点方案，并已上报国家发改委。迎接安顺试验区成立20周年相关工作扎实推进。农村综合改革、国有企业改革改制、政府收支分类改革稳步推进。平坝县集体林权制度改革试点工作顺利完成。组建城市商业银行工作取得实质性进展。着力加快市国资公司和城投公司的改革发展，增强了投融资能力。规范了市区两级之间的土地和城市管理体制。招商引资政策措施进一步完善。与国家开发银行签订了授信贷款额度为16亿元的第二轮合作协议，政银合作深入推进。非公有制经济发展加快，全市非公有制经济增加值占生产总值的比重达50.51%。对外开放取得良好成效，首次组团到香港成功进行了一系列旅游推介和招商引资活动，组织参加了“哈洽会”、东西部合作与投资贸易洽谈会、泛珠经贸洽谈会等对外经贸和招商活动。与苏州、青岛等城市的友好合作全面推进。与九三学社中央开展了“九顺合作”。全年招商引资到位资金31亿元，同比增长45.3%。

五、民主法制和精神文明建设不断加强，维护社会稳定工作扎实推进

自觉接受市人大及其常委会的法律监督和工作监督，自觉接受市政协的民主监督。共办理省人大代表、省政协委员的建议和提案14件，办理市人大代表建议145件，市政协委员提案180件，办复率100%，做到了件件有答复，事事有回音。认真听取各民主党派、工商联和人民团体的意见和建议，积极支持工会、共青团、妇联、科协、红十字会等人民团体依法按章履行职责。自觉接受新闻舆论监督，重视人民群众对政府机关及其工作人员的监督。政务、厂务、村务公开力度进一步加大。扎实开展“五五”普法和法制宣传教育工作，干部群众的法治观念进一步增强。完成了第七届村（居）委会换届选举工作，基层自治组织和社区建设得到加强。

在全市广泛开展了“罗仙精神”、“山鹰精神”和“黄土桥经验”学习宣传活动。评选了“2007感动安顺十大事件、十大人物”，开展了“市树市花”评选活动。“整脏治乱”专项行动获得全省目标考核二等奖，城乡环境得到改善。关岭自治县荣获全国爱国拥军模范单位称号，平坝县、普定县被授予省双拥模范（县）城称号。

加强社会治安综合治理，打击“两抢一盗”等违法犯罪力度加大，禁毒工作三年目标顺利实现，基层矛盾纠纷排查化解体系基本建立，“平安安顺”创建活动扎实推进。信访工作不断加强，群众的合理诉求得到解决。坚持安全发展，强化安全生产监管，有效遏制重特大安全事故，安全生产实现“双降”目标。进一步完善了突发公共事件应急管理体系，处置突发事件的能力得到提高。

六、切实转变政府职能，政府自身建设加快

认真履行政府职能，在继续加强经济调节和市场监管的同时，更加注重搞好社会管理和公共服务。进一步规范了市人民政府工作制度，政府民主决策、科学决策和依法行政的能力和水平得到提高，群众对行政事务知情、参与和监督的渠道进一步拓宽。推进电子政务建设，启动了政府办公信息化建设工作。扎实开展机关作风教育整顿活动，大力纠正部门和行业不正之风，政府机关工作作风进一步转变，行政效能进一步提高。公务员教育、培训和管理进一步加强。坚持不懈地开展反腐倡廉斗争，切实加强对重点环节和重点部位权力行使的制约和监督，治理商业贿赂专项工作扎实推进，政府廉政建设进一步加强。

各位代表，2007年我市经济社会发展取得的成绩，是省委、省人民政府和市委正确领导的结果，是市人大和市政协监督、支持的结果，是各级各部门牢固树立和全面贯彻落实科学发展观和正确政绩观的结果，是全市各族干部群众克服困难，艰苦奋斗，抢抓机遇，真抓实干的结果。在此，我代表市人民政府，向全市广大工人、农民、知识分子、各级干部和离退休老同志，向人民解放军指战员、预备役部队、武警官兵，向人大代表、政协委员，向各民主党派、工商联、人民团体、无党派人士，向所有关心、支持安顺改革开放和现代化建设的各界人士致以崇高的敬意和衷心的感谢！

在肯定成绩的同时，我们也清醒地看到，欠发达、欠开发仍然是我市的基本市情，经济社会发展中还存在一些突出的困难和问题。主要是：经济总量小、实力弱，迅速增加总量同优化结构、提高质量的矛盾突出；农民增收渠道还不多，新阶段扶贫开发任务仍然十分繁重；部分重点领域投资减缓，确保投资合理增长的机制亟待完善；城乡公共服务基础设施薄弱；劳动就业、社会保障、教育、卫生、住房、安全生产、社会治安等方面还有不少亟待解决的问题；各级政府公共服务能力弱，改善民生工作仍有不少需要加强的地方；部分政府工作人员依法行政意识差，依法行政能力不强；一些地方、部门和单位不同程度地存在形式主义、官僚主义现象；发展环境与提速发展的要求还不相适应，等等。对以上问题，我们将高度重视，并继续采取有力措施切实加以解决。

2008年政府工作主要任务

今年是全面贯彻落实党的十七大精神的第一年。我们将迎来改革开放30周年、安顺试验区成立20周年和关岭“顶云经验”30周年。做好今年的工作，保持经济社会发展的良好势头，意义十分重大。

元月中旬以来，我市遭受了50年不遇的低温雪凝灾害，给社会生产和广大人民群众生活造成巨大损失，据统计灾害造成损失约36.15亿元。灾情发生后，我们按照党中央、国务院，省委、省政府和市委的安排部署，及时研究制定应对措施，使全市“科学抗雪凝、切实保民生”工作有序推进。全市上下万众一心，众志成城，奋力拼搏，夺取了抗灾救灾工作的阶段性胜利。灾害期间，市区和各县城没有因灾发生大面积的停电、停水，全市没有出现长时间交通中断和旅客滞留现象，市场货源供应充足，物价稳定，社会秩序良好，人心安定。目前，电力、供水、道路、通信等基础设施全面恢复，农业恢复生产和春耕生产顺利推进，用电负荷和工业生产达到灾前水平，旅游市场全面启动，各项灾后恢复重建工作正加紧展开，为确保全年经济社会又好又快发展赢得了主动。在抗击雪凝灾害中，涌现出许多感人至深、可歌可泣的先进集体、先进个人、先进事迹。实践证明，安顺人民是任何困难都压不倒的。

2008年政府工作的总体要求是：高举中国特色社会主义伟大旗帜，以邓小平理论和“三个代表”重要思想为指导，深入贯彻落实科学发展观，全面贯彻落实党的十七大精神，按照党中央、国务院，省委、省政府和市委的各项工作部署，围绕经济社会发展历史性跨越的奋斗目标，继续解放思想，坚持改革开放，推动科学发展，促进社会和谐，坚持抢抓机遇、好字优先、能快则快，着力优化经济结构和加快转变经济发展方式，大力推进生态文明建设，打造健康长寿之都，千方百计抓重建，竭尽全力保民生，努力实现经济社会又好又快发展，奋力开创改革开放和社会主义现代化建设新局面。

2008年国民经济和社会发展的主要预期目标是：（1）全市生产总值增长12%以上。（2）全社会固定资产投资增长16%。（3）社会消费品零售总额增长16%。（4）财政总收入和地方财政收入分别增长12%。（5）城镇居民人均可支配收入实际增长10%以上，农民人均纯收入实际增长10%以上。（6）城镇新增就业1.2万人，城镇登记失业率控制在4.5%以内。（7）人口出生率和自然增长率分别控制在13‰和6.8‰以内。（8）单位生产总值能耗比上年降低4.29%。（9）主要污染物排放减少2%。

围绕上述总体要求和主要目标，今年将重点抓好以下工作：

一、加快转变发展方式，提高产业竞争力

树立抓产业就是抓发展，抓产业就是抓财政增收，抓产业就是抓改善民生的观念，不断把握发展规律，转变发展方式，优化产业结构，促进产业升级。

（一）紧紧抓住我市被确定为民用航空产业国家高技术产业基地的机遇，大力发展现代装备制造业和特色产业。今年2月，我市被国家发改委确定为全国4个民用航空产业国家高技术产业基地之一。这是我市在加快发展的新形势下一个重大的产业发展机遇，对推进我市经济社会发展具有深远的意义。紧紧抓住和用好这一重大的机遇，科学把握国内外航空产业发展趋势，超前谋划基地发展，把安顺市民用航空产业国家高技术产业基地建设成为军民结合高技术产业集中区，成为全国现代装备制造业的重要基地，成为国内民用航空产业的重要组成部分，成为加快发展的新的增长极。充分发挥政府的宏观指导作用，以市场为导向、以省政府和中航集团签订的合作协议框架为依托、以打造1万亩的核心区域为平台、以国际合作研制4座通用飞机为突破口，加快基地建设步伐，重点发展30座以下通用飞机、航空零部件转包、航空发动机及大型成套产品，加快形成完整的通用飞机工业、营运业、维修培训和综合保障等三条产业链。创新投融资机制，加大对基地基础设施和产业发展的投入，尽快形成生产能力和产业规模。充分发挥基地的示范带动效应，积极争取国家和省的更多重大项目在基地落户实施。继续抓好其他工业园区的发展，加快企业进园区、工业园区化，促进产业聚集、集约生产、规模发展。

积极支持能源工业加快发展，配合做好光照、董箐、马马崖水电站建设。扎实开展乡镇煤矿整合技改，大力实施煤矿标准化建设。围绕农业产业化办工业，大力发展农产品精深加工、民族制药、特色食品等优势产业和特色产业。围绕旅游业发展工业，充分用好“多彩贵州”旅游商品设计大赛和能工巧匠选拔大赛的成果，搞好旅游商品的深度开发，促进旅游商品实现工业化生产、产业化发展。支持西秀区、平坝县、镇宁自治县加快发展白酒产业。在抓好现有工业企业生产经营的同时，积极扶持一批新的工业项目投产、达产，重点抓好烟叶薄片、天马轴承、天台山制药、红星发展不溶性硫、普定超宇200万吨新型干法水泥、黄果树铝业二期技改等项目的实施。确保全市工业增加值增长16%以上。

（二）以旅游业为龙头，大力发展现代服务业。深化中国优秀旅游城市建设，着力提升城市综合素质，加快把安顺建设成为重要的旅游目的地。完成《安顺市旅游业发展总体规划》和《安顺市旅游公路规划》编制工作。大力实施精品战略，加快把黄果树、龙宫两个国家“5A”级旅游景区打造成为世界喀斯特风光游的重要景区和休闲度假胜地；紧紧抓住世界上唯一性的屯堡文化、关岭古生物化石群等特色资源优势，加快把历史文化游培育成为支撑旅游产业发展的重要元素；深入挖掘和包装多姿多彩的民族文化，使民族文化游成为吸引游客的重要旅游项目；大力发展乡村旅游，引导就近游、休闲游，进一步丰富旅游内容。认真搞好旅游线路的设计，重点推出一日游、二日游、三日游精品线路，满足不同游客的需求，让游客游在安顺、享受在安顺、健康在安顺、满意在安顺。

加大旅游基础设施建设力度。大力发展酒店、旅行社、大型旅游购物中心等旅游服务设施，加快旅游信息咨询服务平台建设，提高旅游综合接待能力。加大黄果树“半边街”搬迁工作力度，推进黄果树新城建设。深化旅游管理体制改革，加快黄果树旅游集团公司重组工作；整合屯堡文化旅游资源，组建屯堡文化旅游股份公司。抓好旅游人才培训基地建设，全面提高旅游从业人员综合素质。启动关岭古生物化石群国家地质公园申报国家自然遗产名录和贵州省三叠纪世界地质公园主园区工作，力争把屯堡文化旅游区、关岭花江大峡谷—古生物化石群申报为国家级重点景区。支持紫云自治县开展格凸河申报世界自然遗产（南方喀斯特拓展地）工作。充分利用北京奥运会的机遇，以黄果树瀑布节等旅游节庆活动为载体，整合全市旅游资源，加大整体宣传促销力度，进一步拓展旅游客源市场，提高安顺知名度。力争实现全年接待游客1000万人次以上，旅游总收入75亿元左右。

加快发展第三产业。大力发展交通运输、金融、信息、科技、中介服务等面向生产的服务业。围绕方便群众生活需求，结合推进城镇化进程，加快发展市政公用事业、房地产、物业服务和社区服务等面向民生的服务业。扎实抓好“万村千乡市场工程”，加快发展农资配送服务和农产品收、储、运等面向农村的服务业。全面落实国家和省关于加快服务业发展的各项政策规定，尽快完善促进服务业发展的政策措施，不断优化服务业发展环境。

（三）以促进农业发展农民增收为目标，巩固和强化农业基础地位。认真贯彻落实中央1号文件精神，加强农业基础建设，促进农业发展、农民增收。扎实推进新农村建设，抓好省、市、县三级新农村建设示范工作，确保取得实效。认真抓好粮食生产，继续实施粮食增产工程和超高产示范工程，确保全年粮食总产量稳定在78万吨左右。积极发展油菜、蔬菜、水果、中药材等特色优势作物。抓住国家提高烤烟补助标准的机遇，全面完成21万担烤烟收购任务。做大做强茶产业，培育好茶叶发展重点县。大力发展生态畜牧业，进一步扶持生猪生产，实施好草地生态畜牧业和优质畜产品基地项目，积极推进畜牧业产业化发展，确保畜牧业产值占农业总产值的比重提高1.5个百分点以上。加强农业标准化工作，积极发展无公害、绿色、有机农产品，开展农产品产地认定和产品认证。坚持实施“一乡一特、一村一品”工程，加快培育一批特色明显、竞争力强的专业村镇。大力发展农产品生产、加工、中介、流通等龙头企业，积极发展订单农业，促进农业生产向规模化、商品化和优质、高效的方向发展。积极发展农民专业合作社和农村服务组织。强化农业科技和服务体系建设，推进农业机械化。

坚持开发式扶贫方针，积极推进扶贫开发方式由扶持温饱型向推动发展型转变。积极开展社会扶贫，坚持实施领导干部扶贫联系点和机关对口帮扶制度。实施好114个贫困村“整村推进”，认真做好 “阳光工程”和“雨露计划”培训转移工作，提高劳务收入在农民收入中的比重。

二、加快推进以交通、水利为重点的基础设施建设，切实改善发展条件

加快交通基础设施建设。结合省开展的高速公路网修编工作，继续配合做好黄望高速公路前期工作，争取早日开工建设。配合做好黄果树大桥和黄织铁路建设工作。配合做好厦蓉高速公路清镇至织金段开工建设工作。超前谋划贵昆快速铁路建设相关工作，积极做好安顺至贵阳轻轨项目前期工作。全面完成两六路建设任务。认真组织实施好通乡油路、通村公路和乡镇客运站建设项目。大力发展农村客运，提高农村客运通达深度，实现60% 以上的行政村通客车。推进黄果树机场开展对外合作，加快发展。

围绕解决工程性缺水问题，加强水利基础设施建设。认真实施农村饮水安全工程，解决好农村群众饮水安全问题。推进安西灌区配套工程建设，积极争取并实施好“益民工程”、“滋黔工程”等项目，新增有效灌溉面积5.8万亩。完成烟水配套工程7.6万亩。配合建设好黔中水利枢纽一期工程，做好黄家湾水利枢纽工程前期工作，实施好紫云三岔沟、镇宁麻元、普定窄口等病险水库除险加固工程。积极探索在农村开发地下水用于农业灌溉和饮用的办法，科学合理利用地下水资源。

加快建设“数字安顺”。积极推进互联网、通信网、电视网“三网融合”。以现有网络为基础，加大资源整合力度，加快电子政务统一网络平台建设，完成市政府政务信息传输系统一期工程，促进信息化水平的提高。

三、大力推进城镇化，增强城市对区域经济的辐射带动能力

围绕构建百万人口生态旅游城市的目标，努力在东西拓展、南北贯通、完善功能、开发新区方面有新的进展，加快形成“五区三自治县”的发展框架，力争每年城镇化率提高1.5个百分点以上。

认真做好规划编制工作。按照因地制宜、突出特色、适度超前的思路，完成城市总体规划、土地利用总体规划和城市路网规划修编工作。支持各县开展总体规划修编，提高详规覆盖率。加强规划管理，切实维护规划的严肃性和权威性。

加强以路网为重点的城市基础设施建设。实施二环路建设项目，完成南航路改造、友爱路段拆迁安置等工程，开工建设龙青路、安普路一期、武当路一期等工程，完成新一轮“8·31”项目。推进城市燃气项目的实施，配合做好中缅油气管道项目规划建设工作。加快虹山公园改造工程，启动市级第二行政中心项目前期工作。盘活土地存量，加大土地收储力度，实施连片成规模的城市开发建设。大力实施城市绿化、净化、亮化、美化工程，努力做到四季常绿、季季有花，着力提高城市绿化率、垃圾无害化处理率、污水处理率、燃气使用率、道路人均占有率，进一步完善城市功能。

加大城市管理工作力度。健全市区两级与街道和社区相互衔接、合理分工的城市管理体制，把城市管理工作重心向街道、社区下移，形成全民参与城市管理的新格局，不断提升城市管理工作水平。针对管理效率不高、基础工作薄弱等问题，加强和完善日常管理和基层基础工作，建立经常化管理和责任追究等长效机制，进一步把城市管理工作纳入规范化、制度化轨道。加快建设一支适应我市城市发展需要的规划、建设、管理人才队伍。

推进县城和小城镇建设。继续做好虹阳区申报工作；积极做好平坝县、普定县“撤县设区”工作；以有条件的建制镇和村为基础，加快“镇改办”、“村改居”步伐。把小城镇建设同发展农村二、三产业有机结合起来，增强小城镇承接中心城市功能和带动农村区域经济社会发展的能力。推进户籍制度改革，引导农村人口向城镇集聚。

四、坚持走建设生态文明之路，加强生态环境保护和能源资源节约

牢固树立生态文明观念，打造健康长寿之都，大力推进绿色工业化、绿色城镇化、信息化、知识化和生态化协调发展，努力让安顺山更绿、水更清、天更蓝、空气更清新。

切实加强生态建设。全力推进环城林带一期工程建设，继续抓好退耕还林、天然林保护、珠江防护林体系建设等林业重点工程，完成营造林任务14万亩以上。认真落实国家完善退耕还林的政策措施，进一步巩固退耕还林成果。继续抓好小流域综合治理、“珠治”工程等水土保持生态建设工程，加快水土流失治理步伐。实施好石漠化综合治理试点工程，尽快提高石漠化地区森林覆盖率。加大沼气工程和“改厨、改厕、改圈”工程建设力度。

积极推进节能减排。抓好重点行业、重点企业能耗的控制，实施好安顺电厂脱硫等节能减排重点工程。加快淘汰高耗能、高排放、低效益的落后生产能力，积极开发和推广节约、替代、循环利用资源的先进技术，力争二氧化硫和化学需氧量排放总量稳定下降。大力推进节能节水节地节材，加强资源综合利用，全面推进清洁生产，形成低投入、低消耗、低排放和高效率的节约型增长方式。开展节水型企业和节水型城市创建工作。力争把普定循环经济工业基地申报为省级循环经济试点。强化激励和约束机制，更加注重运用法律手段促进节能减排。

加大环境保护和污染治理力度。加强饮用水源地环境保护，完成集中式饮用水源一级、二级保护区地理界线的实地标定，取缔饮用水源保护区内的直接排污口。加强农村环境保护，切实防治农业面源污染。认真开展第一次污染源普查工作。完成市污水处理厂和平坝县、普定县污水处理厂及贯城河治理二期工程，加快县区污水处理厂和垃圾填埋场前期工作。严格执行地质环境恢复治理保证金征收管理制度，加大地质灾害防治力度。在资源开发中坚持做到护我环境、惜我资源、利我地方、惠我群众，切实防止损害群众利益的问题发生。

五、着力保障和改善民生，加快构建“和谐安顺”

把民生作为政府最大的公务，认真解决人民群众最关心、最直接、最现实的利益问题，努力使全市人民学有所教、劳有所得、病有所医、老有所养、住有所居，不断推进“和谐安顺”建设取得新进展。

优先发展教育。巩固提高“两基”攻坚成果，认真做好“两基”迎“国检”工作，确保镇宁、关岭自治县通过省“两基”复查和“普实”验收。完善农村义务教育经费保障机制，认真落实“两免一补”和提高农村义务教育阶段生均公用经费标准政策，将免费提供教科书的范围扩大到全部农村义务教育阶段学生。从今年秋季起，免除城市义务教育学杂费。积极化解农村义务教育“普九”债务。健全贫困生资助体系。改善农村办学条件，加大农村义务教育阶段薄弱学校改造工程和中西部农村初中改造工程实施力度。做好迎接教育部对安顺职业技术学院的人才培养水平评估工作，加快安顺市第二高级中学新校区建设。大力发展高中阶段教育、职业教育、高等教育，积极发展民族教育、特殊教育、学前教育、远程教育，支持社会力量办学，促进各级各类教育协调发展。扎实做好人才培养、引进和开发工作，努力营造优秀人才脱颖而出的良好环境。

实施积极的就业政策，努力增加城乡居民收入。完善面向所有困难群众的就业援助制度，认真解决好“零就业家庭”和困难家庭就业问题。加强高校毕业生、转业退伍军人、新增就业人员的就业指导，健全面向全体劳动者的职业教育培训制度，建立统一规范的城乡人力资源市场。认真贯彻执行劳动合同法、就业促进法、劳动争议调解仲裁法，进一步规范和协调劳动关系，维护劳动者合法权益。落实最低工资制度和职工带薪年休假制度。确保农民工工资按时足额发放。进一步提高企业退休人员基本养老金水平。认真做好规范公务员津补贴工作，加快推进事业单位收入分配改革。

扩大公共医疗卫生服务。抓好新型农村合作医疗工作，认真落实中央提高补助标准的政策。开展城镇居民基本医疗保险试点，保证参保率达50%以上。进一步提高农民工医疗保险参保率，认真做好服务行业农民工参加工伤保险试点工作。加强农村三级卫生服务网络和城市社区卫生服务体系建设，鼓励建立“惠民医院”、“惠民病房”，为群众提供安全有效、方便价廉的医疗卫生服务。做好重大疾病预防控制工作，切实加强免疫规划工作。建立医患纠纷调处机制，妥善处理医患矛盾。加强食品药品质量安全工作。

进一步健全和完善社会保障体系。积极推进养老保险和失业保险市级统筹，逐步做实养老保险个人账户。提高非公有制企业从业人员、灵活就业人员和农民工的社会保险参保率。坚持应保尽保、分类施保，巩固和提高城市低保的动态管理水平，完善农村低保制度。强化社会保障基金监管，确保基金安全，实现保值增值。进一步重视解决失地农民等特殊困难群众的长远生计问题。发展社会福利事业，鼓励和支持慈善事业发展。

建立住房保障体系。完善廉租住房制度，加强经济适用住房建设和管理，把住房保障和房价稳定列入各级政府目标责任管理。完成廉租住房和经济适用房建设任务，增加普通中低价位、中小套型普通商品住房供应，认真解决城市低收入群众住房困难，努力满足人民群众的住房需求。完善住房公积金制度，进一步提高归集率和使用率。加强农村住房建设规划和管理，实施农村危房改造工程，切实解决农村困难群众住房安全问题。

努力保持物价基本稳定。继续落实好“米袋子”、“菜篮子”行政首长负责制，切实保障粮油和鲜活农产品的供给。加强对市场供求和价格变化的调控监管，依法打击串通涨价、囤积居奇、哄抬物价等违法违规行为，防止部分农产品价格上涨向其他商品蔓延，防止价格过快上涨给低收入群众生活带来过大影响。

集中力量办好市委、市政府确定的“十件实事”：①建设通乡油路261公里、通村公路1000公里、乡镇客运站10个。②实现易地扶贫搬迁500户、2000人；建设农村沼气池20000口；解决农村2.6万户、12万人饮水和饮水安全问题。③减少农村低收入贫困人口27800人；实施农村贫困劳动力就业转移培训16000人，其中“阳光工程”培训8000人，“雨露计划”培训8000人，转移率80%以上。④实施农村薄弱学校改造65个、中西部农村初中改造工程7所；新建20户以上自然村广播电视村村通工程1100座；建成40个村级农民体育健身场地。⑤新建2个县计生服务站，新建、改扩建10个中心乡镇计生服务站和100个村级计生服务室。⑥加快环城林带和中心城区生态景观建设，完成人工造林1万亩。⑦新增就业12000人，其中：完成下岗失业人员再就业4200人，困难人员就业1000人。⑧开展城镇居民基本医疗保险试点，提高城乡居民“低保”标准。⑨开工建设二环路，完成南航路改扩建工程。⑩新建经济适用住房30万平方米，新建廉租住房3万平方米。

六、加快发展各项社会事业，促进经济社会协调发展

认真实施科技发展规划，加快科技进步与创新，提高科技对经济增长的贡献率。积极开展科普工作。大力发展文化事业和文化产业，推进社会主义文化大发展、大繁荣。加强文化基础设施建设，新建或改造300个行政村“农家书屋”，完成1686个农村文化信息资源共享工程。保护和合理开发利用民族民间文化资源，重视非物质文化的传承和保护，建立民族民间文化传承机制。加大文物保护工作力度，抓好第三次全国文物普查工作。继续实施农村电影放映工程。大力推进文化与旅游的结合，促进文化产业加快发展。加大城市历史文化资源的保护和开发力度，积极开展申报中国历史文化名城工作。稳定和降低生育水平，提高出生人口素质，综合治理出生人口性别比升高问题，强化流动人口计划生育服务和管理。加强城乡体育设施建设，抓好村级农民体育健身场地项目的实施，启动安顺市体育馆建设工程。广泛开展全民健身运动，积极组织参加全省首届农民运动会。做好奥运火炬在我市的展示工作。进一步做好宗教、对台、人防、外事侨务、档案、史志、老龄、残疾人等工作。

加强精神文明建设。切实把社会主义核心价值体系融入精神文明建设全过程，巩固全市各族人民大团结的共同思想基础。以“整脏治乱”和公民道德建设工程—“满意在贵州”为重要抓手，大力创建文明城市、文明行业、文明单位、文明村镇。开展创建全国卫生城市工作。在全社会广泛开展“迎奥运、讲文明、树新风”活动，继续开展文化、科技、卫生“三下乡”活动，抓好文化、科技、卫生、法律“四进社区”服务，努力形成崇尚科学文明的良好风尚。扎实推进国防后备力量建设，开展创建“双拥”模范城市工作。

七、进一步深化改革扩大开放，大力营造良好的发展环境

深入推进安顺试验区改革，认真开展迎接安顺试验区成立20周年和“顶云经验”30周年各项工作，继续加大申报国家综合配套改革试点力度。毫不动摇地鼓励、支持和引导非公有制经济发展。把发展非公有制经济作为推进新型工业化、城镇化和市场化的突破口，加强中小企业服务体系建设，落实好市场准入和融资等方面的支持措施，帮助非公有制企业解决发展中遇到的困难和问题，努力消除影响非公有制经济发展的做法和规定，着力营造全社会关心、支持非公有制经济发展的良好氛围，推动非公有制经济大发展。基本完成国有企业改革改制，加快推进集体企业改革改制。完善国有资产监管机制，确保国有资产保值增值。进一步推进市国资公司和城投公司加快发展，做大做强投融资平台。支持有条件的企业上市融资。完成城市信用社组建城市商业银行工作，配合做好邮政储蓄银行安顺分行的组建工作。加强与国家开发银行等金融机构的合作，扩大政银合作领域，拓宽投融资渠道。创新土地管理体制机制，不断提高土地资源的使用效率。认真做好事业单位分类改革工作，继续推进市政公用事业等方面的改革。

深化财政管理体制改革。加快完善公共财政体系，逐步健全财力与事权相匹配的财政体制。着力培育财源，优化财源结构，逐步提高税收收入占财政收入的比重。强化财政预算刚性，依法加强财政监督和税收征管。用好财政增量，调整财政支出结构，新增财力主要用于改善民生和加强政府公共服务。进一步完善建设经济发展先进县区的政策措施，增强县区自我发展能力。支持西秀区搞好省级经济强县建设，支持有条件的县进入省级经济强县行列。

深入推进农村各项改革。认真做好“三改革一化解”工作。完善涉农补贴“一折通”兑现办法。全面推进集体林权制度改革。支持农村金融体制改革和创新，认真做好农业政策性保险工作。深化粮食流通体制、供销社体制和农村小型基础设施产权改革。稳定和完善土地承包关系，切实保障农民土地权益，稳妥规范地推进农村土地承包经营权流转，支持有条件的地方发展多种形式的适度规模经营。

进一步扩大对内对外开放。在完善经济发展基础条件的同时，努力营造良好的法制、政策、市场、服务、信用环境，在更宽的领域、更高的层次，全方位开展对外交流与合作。进一步强化和规范行政服务中心服务职能，全面深入开展“群众评议机关”、“民主评议行风”等活动，提高服务效率。大力实施“引进来”和“走出去”战略，特别要围绕民用航空产业国家高技术产业基地和园区的产业发展规划，有针对性地开展国际国内招商，争取在引进战略投资者方面见到实效。进一步加强招商引资项目库建设，着力在航空产业、装备制造业、旅游开发、城市建设、企业重组、农产品精深加工等方面组装一批大项目向外推出，有重点地加强对长三角、珠三角等资本、技术密集地区的“目的地”招商，扩大招商引资规模，实现招商引资到位资金45亿元以上。加强项目调研、编制和储备工作，加大项目前期工作力度，提高项目质量。坚持招商与选商相结合，引商与引资相结合，引资与引智相结合，使引进的项目符合国家产业政策和环保要求、符合生态文明建设方向，有利于扩大就业、增加群众收入和优化产业结构。建立健全招商引资项目落实责任制，加强对重点项目、重点投资企业的跟踪服务，提高招商引资项目签约率、开工率、投产率。深入推进与苏州、青岛等城市的友好合作，巩固和扩大“九顺合作”。

八、大力加强民主法制建设，促进社会公平正义

加强民主法制建设。自觉接受市人大及其常委会的工作监督和法律监督，自觉接受市政协的民主监督。认真听取各民主党派、工商联和无党派人士的意见和建议，支持工会、共青团、妇联、红十字会等人民团体依法按章履行职责，开展工作。依照有关法律的规定接受司法机关实施的监督。高度重视新闻舆论监督和人民群众监督。加强政府层级监督，充分发挥监察、审计等专门监督的作用。发展基层民主，完善基层群众自治制度，推动城乡社区建设。贯彻落实政府信息公开条例，深入推进政务、村务、厂务公开和公用部门办事公开，增强政府工作的透明度，扩大人民群众对行政事务的知情权、参与权、表达权和监督权。做好法律服务和法律援助工作。继续抓好“五五”普法和法制宣传教育，在全社会形成自觉守法用法的良好氛围。全面落实民族区域自治制度，促进民族地区经济社会发展，巩固和发展平等团结互助和谐的社会主义民族关系。

切实维护社会稳定。健全党委领导、政府负责、社会协同、公众参与的社会管理格局。正确处理人民内部矛盾，认真开展基层矛盾纠纷的排查和化解，做好人民群众来信来访工作，继续落实领导干部接访制度。高度重视在企业改制、征地拆迁、资源开发、移民安置等方面损害群众利益的问题，切实维护人民群众的合法权益。强化公安系统“三基”工程建设，继续搞好基层派出所、乡镇法庭和乡镇司法所建设。大力创建“平安安顺”，坚决打击各类刑事犯罪和经济犯罪，特别是黑恶势力，进一步加强社会治安综合治理。高度警惕和防范敌对势力的破坏活动，坚决维护国家安全。

高度重视安全生产。认真落实安全生产责任制，健全安全生产保障体系，督促推动企业严格履行安全设施建设“三同时”规定，切实做到关口前移、重心下移。继续深入开展重点行业、重点领域的隐患排查治理和以煤矿、道路交通等为主要内容的安全专项整治，着力解决事故多发行业和领域的突出问题，坚决遏制重特大事故发生，努力实现安全生产“双降”目标。

九、统筹兼顾，抓好灾后恢复重建工作

紧紧围绕全年的工作部署，坚持目标不变，任务不减，一手抓发展第一要务不动摇，一手抓灾后恢复重建工作不放松，确保大灾之年群众的生活水平不降低，经济社会发展的速度不减慢。统筹调配人力、物力、财力，科学规划，合理设计，继续深入扎实推进灾后恢复重建各项工作。进一步安排好受灾群众的生产生活，抓好受灾民房的修复和重建，确保损房户房屋全面修复，倒房户6月底前搬进新居。加快修复重要基础设施，加大对工农业生产的支持和扶持力度，进一步振兴旅游市场，努力夺回灾害造成的损失。严格救灾款物的使用和监管，确保救灾款物全部用在灾后恢复和重建家园上。高度重视雪凝天气可能引发的次生灾害，切实做好地质灾害、森林防火、防汛抗旱、公共卫生等方面的安全防范工作。要从这次雪凝灾害中认真总结经验教训，加强电力、交通、通信等基础设施建设，提高抗灾和保障能力；进一步完善应急体系和机制，提高预防和处置突发事件的能力；加强对现代条件下自然灾害的预测预报预警，提高防灾救灾减灾能力。抓住并用好国家和省加大灾后恢复重建支持力度的机遇，积极争取上级的更大投入，推进经济社会加快发展。

加强政府自身建设

按照建设服务政府、责任政府、法治政府和廉洁政府的要求，深化行政管理体制改革，着力转变职能、理顺关系、优化结构、提高效能，努力使政府工作更好地符合广大人民群众的根本利益和普遍意愿，让人民群众满意。

第一，坚持转变政府职能，建设服务政府

在全面履行经济调节、市场监管、社会管理和公共服务职能的基础上，把更多的精力和资源用在加强社会管理上，用在提高公共服务水平上。加快推进政企分开、政资分开、政事分开、政府与市场中介组织分开，把不该由政府管理的事项转移出去，把该由政府管理的事项切实管好，从制度上更好地发挥市场在资源配置中的基础性作用，更好地发挥公民和社会组织在社会公共事务管理中的作用。建立健全公平公正、惠及全民、水平适度、可持续发展的公共服务体系，推进基本公共服务均等化。健全部门间协调配合机制，明确部门责任，确保权责一致。坚持简政放权，分级负责，把更多的权力下放给基层政府，充分保护和调动各级加快发展的积极性、主动性和创造性。

第二，坚持勤政为民，建设责任政府

坚持深入基层、深入群众，始终把全部心思放在发展上，把所有精力放在为民致富、为民谋利上，放在关心群众冷暖上。真心诚意为群众办实事办好事，把实现好、维护好、发展好最广大人民群众的根本利益作为政府工作的出发点和落脚点，对党和人民的事业始终充满热情、激情、真情，对工作尽力、尽职、尽责、尽心。把执政为民的要求融入到思想感情里，体现在政策措施上，落实到具体工作中，多干打基础、利长远的事。健全监督机制，强化责任追究，切实做到有权必有责、用权受监督、侵权要赔偿、违法要追究。加强政府效能建设，推行政府绩效管理和行政问责制度，提高政府执行力和公信力。

第三，坚持依法行政，建设法治政府

全面贯彻落实行政许可法，严格按照法定权限和程序行使职权、履行职责。加强和改善行政执法，不断提高执法水平。坚持科学民主依法决策，完善公众参与、专家论证和政府决策相结合的决策机制，健全和完善重大问题集体决策、专家咨询、社会听证和决策评估等制度，全面提高政府决策水平。加强规范性文件制定工作，提高规范性文件质量。健全行政执法体制，加快建立一支政治合格、纪律严明、业务精通、作风过硬的行政执法队伍。

第四，坚持廉洁从政，建设廉洁政府

严格执行党风廉政建设责任制，加快建立健全教育制度监督并重的惩治和预防腐败体系，进一步推进廉政建设，拓展从源头上防治腐败的工作领域。建立健全决策权、执行权、监督权既相互制约又相互协调的权力结构和运行机制，着力抓好制度的执行，做到用制度管权、按制度办事、靠制度管人，确保权力不被滥用。深入贯彻公务员法，加强公务员队伍建设，对政府系统干部严格要求、严格教育、严格管理、严格监督，坚决杜绝“中梗阻”和“吃拿卡要”等现象，切实转变作风，提高办事效率，树立公务员的良好形象。

各位代表，做好今年的政府工作，任务艰巨，意义重大。让我们更加紧密地团结在以胡锦涛同志为总书记的党中央周围，高举中国特色社会主义伟大旗帜，以邓小平理论和“三个代表”重要思想为指导，深入贯彻落实科学发展观，认真学习贯彻党的十七大精神，在市委的领导下，凝聚全市各族人民的智慧和力量，抢抓机遇、加快发展，同心同德、扎实工作，奋力提速发展、实现黔中崛起，为开创改革开放和社会主义现代化建设新局面而努力奋斗！
